# Randomised, placebo-controlled, double-blinded, four-way crossover trial to demonstrate the comparative pharmacodynamic equivalence of a non-invasive diagnostic test for adrenal insufficiency in a healthy population: the STARLIT-2 study protocol

**DOI:** 10.1136/bmjopen-2024-094830

**Published:** 2024-12-22

**Authors:** Kathryn L Date, Kathleen Baster, Sharon L Caunt, Judith Cohen, Miguel Debono, Jane Fearnside, Trevor N Johnson, Richard J Ross, Rosie N Taylor, Charlotte J Elder

**Affiliations:** 1 Hull Health Trials Unit, University of Hull, Hull, UK; 2 Statistical Services Unit, The University of Sheffield, Sheffield, UK; 3 Academic Directorate of Diabetes and Endocrinology, Sheffield Teaching Hospitals NHS Foundation Trust, Sheffield, UK; 4 Sheffield Centre for Health and Related Research (SCHARR), School of Medicine and Population Health, The University of Sheffield, Sheffield, UK; 5 Translational Sciences Group, Certara UK Limited, Sheffield, UK; 6 Clinical Medicine, School of Medicine and Population Health, The University of Sheffield, Sheffield, UK

**Keywords:** Adrenal disorders, Clinical Trial, Paediatric endocrinology, Randomised Controlled Trial

## Abstract

**ABSTRACT:**

**Introduction:**

Cortisol is an essential stress hormone and failure of its production, known as adrenal insufficiency (AI), is associated with significant mortality due to adrenal crisis. The Short Synacthen Test (SST) is the current diagnostic test of choice for AI, but it is both invasive and resource intensive. Globally, there is an unmet need for a non-invasive, cost-effective test. A novel formulation, Nasacthin, has been developed, which can be delivered intranasally, with the resultant glucocorticoid levels measured in saliva instead of blood. The Salivary Test of Adrenal Response to Liquid Intranasal Tetracosactide (STARLIT-2) study aims to clinically validate the Nasacthin test in healthy volunteers.

**Methods and analysis:**

STARLIT-2 is a randomised, placebo-controlled, double-blinded, four-way crossover trial. 32 healthy adults and children will be randomised to receive each of four study drugs (Synacthen, Nasacthin and their respective placebos) over four study visits (one per visit). Paired blood and saliva samples will be collected from participants at baseline, and then at 30, 60, 90 and 120 min after drug administration. Additional salivary samples will be collected at 180, 240, 360 and 480 min after drug administration. The primary outcome measures are to compare the mean serum cortisol at 30 min after Synacthen or Nasacthin dose, with a view to determine non-inferiority; and to compare the mean change from baseline in serum cortisol at 30 min after active and placebo doses of both Synacthen and Nasacthin, aiming to demonstrate superiority of active over placebo. In addition, the proportion of participants for which Nasacthin produces a rise above a preset serum cortisol threshold at 30 min will be determined, with the negative per cent agreement with the SST calculated using the SST as the reference standard.

**Ethics and dissemination:**

The study and its amendments have been reviewed and approved by South Central–Hampshire A Research Ethics Committee. Results will be disseminated in peer-reviewed journals and conference presentations, and feedback to trial participants will be facilitated following consultation with patient and public involvement and engagement groups.

**Trial registration number:**

ISRCTN62724177

**STRENGTHS AND LIMITATIONS OF THIS STUDY**

- Robust study design that will produce data as agreed with the Medicines and Healthcare products Regulatory Agency (MHRA) to support the regulatory approval of Nasacthin as part of a series of related studies.
- Direct comparison of Short Synacthen Test and Nasacthin test (participants were dexamethasone suppressed in previous development studies), which will gather data on both children and adults (both men and women, as no women were included in previous development studies).
- Will gather data on pharmacodynamic elements in addition to the main outcomes and safety.
- Small participant numbers, meaning the study is not powered to investigate any potential impact from factors such as ethnicity, body mass index, etc.
- No data on effects of coryzal illness, hay fever, etc which could potentially influence the absorption of nasal drug, or on children <4 years of age (unable to participate due to salivary collection techniques).

## Introduction

 Adrenal insufficiency (AI) describes the inability of the adrenal glands to produce an adequate amount of the essential stress hormone, cortisol. AI is classified as either primary (adrenal), secondary (pituitary) or tertiary (hypothalamic, mainly adrenal suppression secondary to prolonged, high-dose exogenous steroid use), dependent on its origin.^1^

Diagnosis of AI is often delayed, due to presentation with non-specific symptoms, leading to an increased risk it will manifest as an episode of adrenal crisis, which carries an associated 6% mortality rate.^2^ The most commonly used and recommended test for diagnosing AI in current clinical practice is the Short Synacthen Test (SST).^3^,^5^ The SST involves either intravenous or intramuscular injection of Synacthen (tetracosactide), a synthetic analogue of adrenocorticotropic hormone (ACTH), consisting of the N-terminal 24 amino acids (1–24), followed by blood sampling at 30±60 min to quantify the serum cortisol response. Requests for SSTs are increasing across all healthcare sectors,^5 6^ likely due to rising concerns around the iatrogenic nature of tertiary AI and increased prescription of glucocorticoid therapies that suppress adrenal function. However, the test is time, labour and resource intensive, requiring skilled personnel and a medical day care admission. It also requires cannulation (or administration of intramuscular Synacthen) and venous sampling, which may be distressing and painful, especially for children. There is an unmet need for a less invasive, less resource-intensive and therefore more cost-effective test to diagnose AI.

Intranasal administration is a minimally invasive technique that can be used to effectively deliver drugs, with the advantages of avoidance of first pass metabolism, good absorption due to the richly vascular nasal mucosa and a rapid onset of action.^7^ Furthermore, minimal training is required to administer the drugs and use of needles can be avoided. Current evidence suggests that the side effects of nasal drugs are few, generally attributable to the drug itself rather than the method of delivery, and resolve rapidly.

The Nasacthin test, which uses an intranasal formulation of tetracosactide (the active pharmaceutical ingredient (API) in Synacthen), has been developed to address this unmet need. Within the Nasacthin formulation, tetracosactide is coupled with chitosan, a mucoadhesive agent, in order to aid nasal absorption. This can be administered nasally via a spray, with the resultant glucocorticoid levels measured in saliva samples to quantify the adrenal response.

Salivary glucocorticoid estimation is growing in popularity due to the advantages of non-invasive sampling and close correlation between salivary cortisol and cortisone, and biologically active free serum cortisol. Salivary cortisol is a validated, well-established alternative to invasive glucocorticoid sampling, and is recommended as a first-line diagnostic test for Cushing’s syndrome.^8^ It has also been investigated following Synacthen stimulation in healthy volunteers and patient populations,^9 10^ and specifically following Nasacthin administration in healthy adult males and children.^11^ However, it is salivary cortisone that has emerged as the preferred salivary biomarker as, due to rapid metabolism of salivary cortisol to cortisone, it is the more abundant glucocorticoid and gives a closer reflection of serum cortisol levels, with better sensitivity at low serum cortisol levels.^10 12 13^ A recent prospective diagnostic accuracy study showed that the waking salivary cortisone level was a strong predictor of the serum cortisol response to Synacthen in the SST.^14^

To date, four pharmacokinetic-pharmacodynamic studies (PK-PD)and a reproducibility study have been completed,^11^ in which a total of 70 doses of Nasacthin have been administered to healthy adult males and children. All participants were dexamethasone suppressed to allow quantification of ACTH_(1–24)_ for PK analysis. These studies found Nasacthin to be reliably absorbed and well tolerated, and importantly demonstrated a comparable plasma cortisol response to the intravenous Synacthen test, and that 60 min is the optimal timing of salivary glucocorticoid sampling following Nasacthin.^9^

The Salivary Test of Adrenal Response to Liquid Intranasal Tetracosactide (STARLIT-2) study aims to collect data to support the clinical validation of the Nasacthin test, by comparing its PD performance to the current standard Synacthen test in a healthy population. The closely linked STARLIT-3 study (due to start recruitment in Winter 2024) will assess its diagnostic performance in an AI patient population, again using the SST as the reference standard.

## Methods and analysis

The study methodology is reported in accordance with the Standard Protocol Items: Recommendations for Interventional Trials reporting guidelines.^15^

### Study aims and objectives

The STARLIT-2 study primarily aims to demonstrate non-inferiority in serum cortisol response following administration of nasal tetracosactide (Nasacthin) to the response following intravenous tetracosactide (Synacthen) administration. In addition, the study will seek to confirm the superiority of active treatments (Nasacthin and Synacthen) over their respective placebo comparators.

As secondary objectives, the study will examine the salivary cortisone response following Nasacthin stimulation with the aim to demonstrate that this is non-inferior to the response following adrenal stimulation with Synacthen, as well as looking at the non-inferiority of the serum cortisol response using change from baseline values instead of absolute values. The study will collect safety data on the use of Nasacthin in healthy volunteers, and will explore the acceptability, usability and tolerability of the Nasacthin test to participants and healthcare professionals. A further exploratory objective will compare the serum cortisol, salivary cortisol and salivary cortisone response at 30 and 60 min after drug administration to explore the relationship of the glucocorticoids in the dynamic phase following administration.

### Study design

STARLIT-2 is a randomised, placebo-controlled, double-blinded, four-way crossover trial conducted in healthy adults and children across two sites, Sheffield Teaching Hospitals NHS Foundation Trust (Sheffield, UK) and Sheffield Children’s NHS Foundation Trust (Sheffield, UK).

### Study population

The study aims to recruit healthy men, women and children aged 4–69 years, with the adult population defined as participants aged 16 years and above. In accordance with Medicines and Healthcare products Regulatory Agency (MHRA) scientific advice, the trial sample size target is 32 participants, who will not be dexamethasone suppressed, completing four study visits. The study population will comprise at least 10 participants of each sex, and 20–50% (n=6–16) participants will be children, as requested by the MHRA. The primary target group for the Nasacthin test, if successfully adopted, will be children, who will particularly benefit from it being a non-invasive test and who are particularly susceptible to the development of tertiary AI.

### Eligibility criteria

Eligibility to participate in the trial will be confirmed by the site principal investigator or appropriately delegated clinician, and will be determined in accordance with the exclusion criteria listed in box 1.

Box 1 **Trial Exclusion Criteria**

1. Known adrenal insufficiency, Cushing’s syndrome or any other adrenal or pituitary gland disorder.
2. Ongoing pregnancy.
3. Use of oestrogen-containing hormonal contraception/hormone replacement therapy.
4. Known condition requiring daily administration of a medication that interferes with the metabolism of glucocorticoids, including all oestrogens, opioids, oral antifungals and loperamide.
5. Currently prescribed antiepileptic medication, such as sodium valproate, phenytoin, clonazepam, nitrazepam, phenobarbital or primidone.
6. Known and active protein losing disorders, for example, enteropathy or nephrotic syndrome.
7. Known clinical or biochemical evidence of hepatic or renal disease.
8. Regular inhaled, topical, nasal, ocular, rectal, oral or intra-articular steroids for any indication in the last 3 months.
9. Current uncontrolled active infection.
10. Body mass index (BMI) >35 kg/m^2^ (or BMI>3 SD scores (SDS) above the mean for age and sex if <16 years).
11. Known or suspected alcohol dependence or drug misuse.
12. Current smoker or vaper (or within 6 months of cessation).
13. Recent (within the last 1 week) liquorice ingestion (preparations containing glycyrrhizic acid only).
14. History of known salivary gland or oral mucosa pathology or who are unable to produce a suitable salivary sample (eg, as a consequence of drugs that cause dry mouth).
15. Previous severe allergic reaction or anaphylaxis, or adverse reaction to any antigen of adrenocorticotropic hormone (ACTH) or Synacthen.
16. Participation in another clinical trial of an investigational or licensed drug within the last 3 months, unless it is a clinical trial of the same investigational medicinal product (IMP) (ie, Nasacthin), in which case only a 7-day washout period applies.
17. Unable to comply with the requirements of the protocol.
18. Any other significant medical or psychiatric conditions that in the opinion of the investigator would preclude participation in the trial.

Exclusions for nasal IMP visits only (visit should be rescheduled):

1. Coryzal symptoms within the last week.
2. Heavy nosebleed within the previous 48 hours.

### Sample size

A sample size of 30 participants was calculated as required to provide >90% power for the non-inferiority primary objective, assuming a non-inferiority margin of −88 nmol/L (equivalence limits of serum cortisol ±88 nmol/L) (as determined from previous work^11^ but with allowance for additional variation in baseline cortisol due to the absence of dexamethasone suppression) and a conservative significance level of α=2.5% (actual alpha to be defined by the truncated Hochberg procedure as part of a hierarchical testing procedure). The same sample size will have >90% power to show a negative percent agreement (NPA) >77.5% assuming a conservative α=2.5% and a one-sided exact test, and >90% power for the tests of superiority assuming a clinically relevant difference in serum cortisol change from baseline of 200 nmol/L and an SD of up to 70 nmol/L and α=5%.

The overall sample size was increased to 32, to allow for up to two participants who do not meet the requirement of a rise above threshold following Synacthen (or are excluded from the trial analysis for some other reason; eg, incomplete data).

Based on experience from previous studies, a dropout rate (including need for repeat visits in willing participants; eg, for cannula failure) of 33% in children and 20% for adults has been factored into the calculations, and therefore the overall recruitment target is 44 participants.

### Recruitment and consent

Healthy volunteers will be recruited via targeted emails to staff and/or students within the hospital Trusts and the University of Sheffield, posters displayed in high-throughput areas and endocrinology clinics at both hospital Trusts, and Trust and University communications and social media platforms inviting expression of interest in participation.

Study-specific, age-appropriate participant information can be accessed online on the study website (https://hhtu.hull.ac.uk/starlit-2/#tab-1564), or will be provided by the research team in response to expression of interest. A parent or legal guardian must express interest on behalf of any potential child participants. A specific Participant Information Leaflet has been developed for parents/legal guardians, and children will receive their own version based on their age (either 5–9 or 10–15 years). The study has also been designed so as to be inclusive of any potential participants with learning disabilities who may wish to take part and have capacity to consent (in accordance with the Mental Capacity Act 2005). An ‘Easy Read’ version of the Participant Information Leaflet has been developed to aid their understanding of the study.

Once eligibility is confirmed, informed consent will be received by an appropriately trained and delegated study clinician or research nurse prior to the first study visit. Consent for participants under 16 years of age will be provided by a parent or legal guardian with confirmed parental responsibility. Current versions of the consent forms can be found in onlinesupplemental materials 1  2. Children will be asked to sign an assent form if deemed developmentally appropriate.

The consent process will primarily be completed using remote e-Consent via DocuSign, although postal and face-to-face options are available if required. Consent will be taken during a real-time phone or video call discussion, and the participant’s identity will be checked at the first face-to-face study visit.

Participants will be enrolled in the study database and a unique sequential participant ID number will be generated for each participant. Copies of all completed consent forms will be uploaded into the study database to enable central remote monitoring of the consent process.

### Randomisation

All enrolled participants will be randomised sequentially via the purpose-built secure web-based data capture system with integrated randomisation function held on REDCap Cloud (RCC; www.redcapcloud.com). This is a four-way crossover trial, in which participants will be randomised to receive a single dose of each of four study investigational medicinal products (IMPs) (one per visit—see box 2) in a predetermined random sequence.

Box 2 **Trial investigational medicinal products (IMPs)**

**TEST IMPs**

- Active intranasal tetracosactide

500 µg Nasacthin (nasal tetracosactide with chitosan)

0.1 ml administered to each nostril (total volume 0.2 ml)

- Placebo intranasal tetracosactide

Nasacthin minus tetracosactide

0.1 ml administered to each nostril (total volume 0.2 ml)

**COMPARATOR IMPs**

- Active intravenous tetracosactide

250 µg Synacthen (145 µg/m^2^ for paediatric participants)

1 ml or suitably reduced volume based on 145 µg/m^2^ for paediatric participants

- Placebo intravenous tetracosactide

0.9% sodium chloride

1 ml bolus or equivalent reduced volume as per calculation for active IV dose for paediatric participants

Previous studies demonstrated that 500 µg Nasacthin provided a comparable plasma cortisol response to the intravenous Synacthen test at 60 min, the optimal timing of salivary glucocorticoid sampling following Nasacthin.^11^ Therefore, this dose will be used for all participants during the trial, with 0.1 mL administered to each nostril. The same volume will be administered of the nasal placebo, with identical constitution to Nasacthin minus the tetracosactide (API).

Adult participants will receive 250 µg Synacthen (1 mL), as per normal practice for the SST, while the paediatric dose of intravenous Synacthen is 145 µg/m^2^, as recommended in the British National Formulary for Children.^16^ The intravenous placebo to be used in the study will be 0.9% sodium chloride, which will be stored under refrigerated conditions to mimic Synacthen storage conditions and therefore avoid unintentional unblinding of study participants.

### Blinding

While the nature of the visit (ie, nasal or intravenous) cannot be blinded, the respective active and placebo treatment allocations will be concealed from the trial investigators, site research teams and trial participants. Site pharmacy teams and an unblinded research nursing team at each site will be unblinded to intervention allocation for IMP preparation and administration purposes. Site pharmacies will be notified of unblinded randomisation allocation via email or using an access-restricted electronic case report form (eCRF), while site research teams can access a blinded sequence to enable appropriate preparations to be made for either intravenous or nasal IMP visits.

### Study procedures

**Figure 1:**
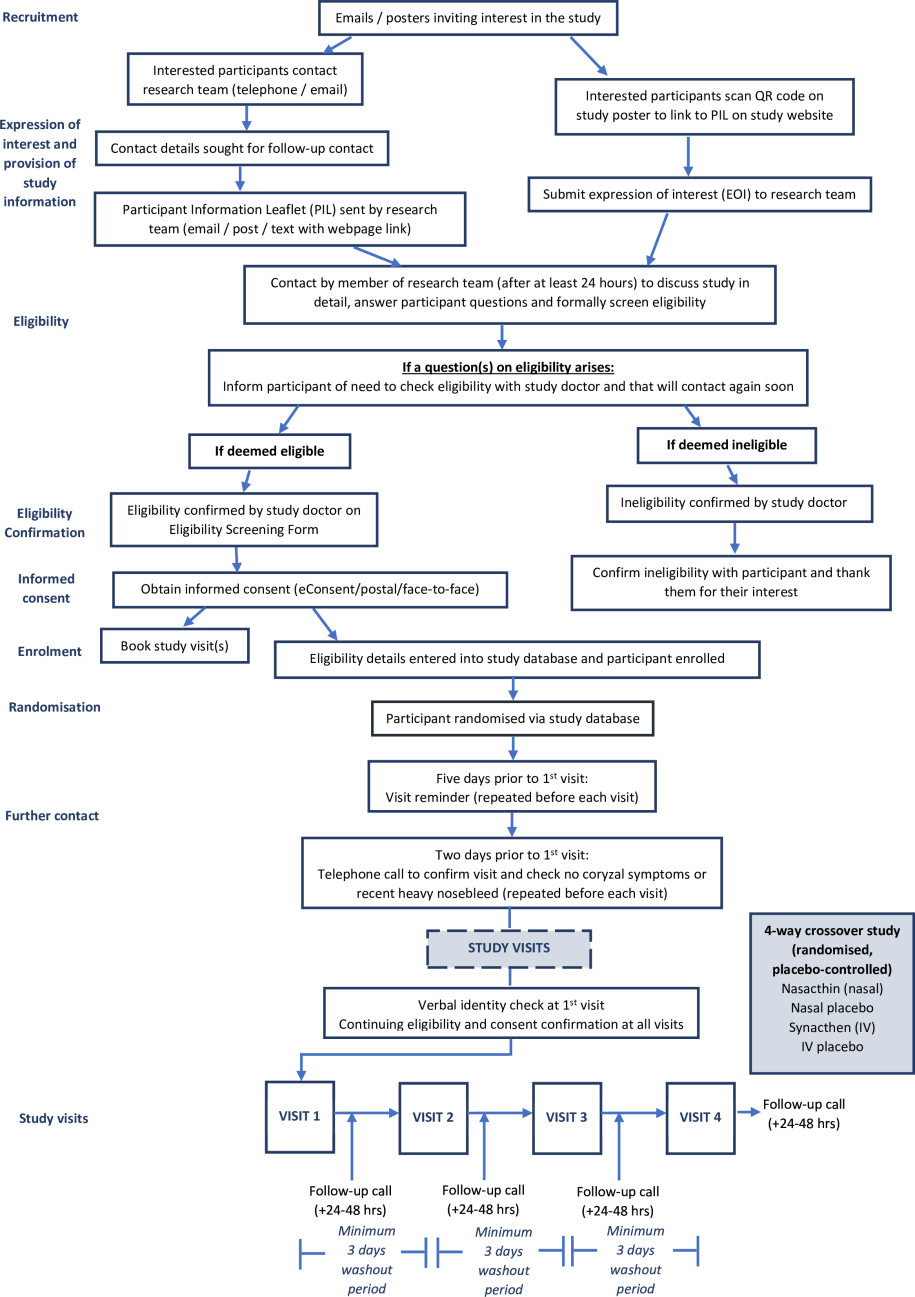
Flow diagram of study procedures. IV, intravenous.

Study procedures are outlined in figure 1. Participants will attend four face-to-face visits with a minimum 3-day washout between visits to allow for sufficient elimination of any previous tetracosactide received (calculated from previous PK data). Eligibility and ongoing consent will be reconfirmed at the start of each visit.

Participants will be contacted at 5 and 2 days prior to each visit to confirm attendance and perform essential checks around visit-specific exclusions. Participants will be asked to abstain from alcohol consumption and recreational drug use for 24 hours and liquorice ingestion within the last week (preparations containing glycyrrhizic acid only), while any nasal visits will be rescheduled if a participant has experienced coryzal symptoms in the last week, or a heavy nosebleed in the previous 48 hours, due to the unknown effect on nasal absorption.

Study visits will commence between 08:00 and 09:00, in order to obtain samples for baseline glucocorticoid determination while cortisol levels are near their physiological peak. At the first study visit, basic demographic data will be collected, and height and weight will be measured. The height and weight measurement will be repeated at subsequent visits for any children who have more than 3 months between visits.

At the start of each visit, a set of baseline clinical observations (temperature, pulse rate, blood pressure and respiratory rate) will be taken to confirm the participant is well, and all participants of childbearing potential will require a confirmed negative pregnancy test before study activities can commence at each visit, due to the potential for altered cortisol levels, and in accordance with the recommendation that Synacthen should not be administered in pregnancy.^17^

Following intravenous cannulation, participants will rest supine for a minimum of 20 min. Participants will be requested to rinse their mouths thoroughly with water 10 min prior to commencing sample collection, after which a pair of baseline blood and saliva samples will be collected. All blood samples will be taken from the indwelling cannula and all saliva samples (minimum 1 mL per sample) will be collected by passive drool into a Saliva Collection Aid (Salimetrics, Pennsylvania, USA).

Whole blood samples will be collected in serum separating tubes, and will be gently mixed and left to clot for 30–60 min at room temperature, before centrifuging at 1300 *×g* for 10 min. Resulting serum will be aliquoted into cryovials for storage. Saliva samples may be centrifuged at 1000 rpm to reduce bubbles if necessary. All samples will then be transferred to −80°C freezer storage prior to shipment to a central laboratory for analysis.

Study IMP will be administered immediately after baseline sample collection, in accordance with the randomised sequence allocation. Test IMP (Nasacthin/nasal placebo) will be administered via a primed intranasal Mucosal Atomisation Device (DART300, Pulmodyne, Indiana, USA) (0.1 mL to each nostril), while intravenous comparator IMP (Synacthen or sodium chloride 0.9%) will be administered as a slow bolus (1 mL or volume as per 145 µg/m^2^ calculation) via the indwelling cannula.

Further paired blood and saliva samples will be taken at the following times (IMP administration at 0 min): 30, 60, 90 and 120 min. Participants will be asked not to eat or drink anything, other than water, until after the 120 min samples have been collected, although children are permitted a small snack immediately after a sample collection if necessary, provided viable 60 min samples have already been collected.

Additional saliva sampling is then required at 180, 240, 360 and 480 min after IMP administration, and this can be completed at the participant’s home/place of work (or at the site if preferred). Eating and drinking is allowed between sample collection times for these later timepoints, but ideally not within the 30 min before a sample collection, and participants should rinse their mouth thoroughly with water at 10 min prior to taking each sample. Participants will be provided with an instruction leaflet with the target sample collection times entered by the research team. Participants will be asked to record the actual sample collection times against the target times, and then to return these samples to the site team, either via Royal Mail Safebox, participant self-return or courier. On receipt, all samples will be transferred to −80°C freezer storage. All participants will receive a follow-up telephone call 24–48 hours (or next available working day) after each visit to check for the emergence of coryzal symptoms or other adverse events (AEs).

### Outcomes and assessments

Participant study samples will be shipped in batches to a central laboratory, where they will be analysed using liquid chromatography with tandem mass spectrometry (LC-MS/MS) assays to determine the levels of serum cortisol, salivary cortisol and salivary cortisone present at each timepoint.

Mean serum cortisol concentrations measured at baseline and at 30 min following stimulation with Nasacthin and Synacthen, as well as mean change from baseline, will be compared for non-inferiority, and will also be used to determine the proportion of participants for which the Nasacthin test produces a rise above a preset threshold, out of those for whom the Synacthen test also produces a rise above the threshold. The mean change from baseline in serum cortisol concentration at baseline and 30 min after Nasacthin and Synacthen stimulation will also be separately compared against their respective placebo drugs to determine superiority.

Mean serum cortisol concentrations and mean change in serum cortisol from baseline after Nasacthin and Synacthen stimulation will additionally be compared using values obtained at baseline and 60 min after drug administration, while mean salivary cortisone concentration and mean change from baseline in salivary cortisone will be similarly compared using the baseline and 60 min after Nasacthin and Synacthen administration timepoints.

Although the aim is to attain a complete set of glucocorticoid results for all samples for all participants across the four study visits, it is not essential in this study for a participant to provide a viable sample for every timepoint at each individual visit. It is, however, vital that those samples critical to the determination of the main study endpoints should be collected in order to ensure sufficient data for the primary and secondary analyses. The baseline and 30 min serum samples are crucial for the primary non-inferiority analysis, for the calculation of the NPA and for the comparisons between active and placebo treatments. The baseline, 30 min and 60 min serum samples are necessary for the secondary non-inferiority analyses, and the baseline and 60 min saliva samples are required to define the salivary cortisone response. The remaining salivary samples will be used for the additional PD analysis to calculate the elimination of cortisol; the minimum requirement for this is the 120 min saliva sample, plus two other samples from the later timepoints up to 480 min.

Individual visits will be repeated if viable key samples for endpoint determination are not obtained (eg, in the instance of cannula failure after IMP administration; blood contamination in saliva samples; unable to produce sufficient saliva for primary samples due to a temporary reason). In this instance, the same IMP will be administered at consecutive visits to ensure the randomised IMP sequence is maintained.

Participants will be closely monitored for any AEs occurring after IMP administration, particularly in the first 30 min, as this is when the majority of hypersensitivity reactions would be expected to occur. A follow-up telephone call 24–48 hours (or next available working day) after will be used as a safety check for any AEs occurring. All AEs, serious adverse events (SAEs) and suspected unexpected serious adverse reactions (SUSARs) will be collected in the study database and coded using the Medical Dictionary for Regulatory Activities (MedDRA), and reported as appropriate.

Participants will be asked to complete a non-validated questionnaire at the end of each visit to explore the acceptability and tolerability of IMP received at that visit and associated study sampling procedures. An additional final questionnaire at the end of their study completion will ask about their overall acceptability and tolerability, comparing both the nasal and intravenous tests.

Towards the end of their involvement in the study, participants will also be invited to take part in an optional one-to-one interview to gain a more in-depth understanding of their views on the acceptability and tolerability of the tests. A purposive sample of those who have given consent to be contacted will be used, with sampling criteria including age and gender, and whether all visits and study sampling were completed. At the end of study recruitment, participating healthcare professionals will be asked to complete an anonymous questionnaire on the comparative acceptability of administration of the two tests.

### Data collection and management

The main study database will be developed and managed by Hull Health Trials Unit (HHTU; University of Hull, Hull, UK) within the HHTU instance of commercial online data capture and randomisation system (RCC). The database will be built and validated according to study-specific requirements with both automated and manual checks to monitor data quality and completeness according to a sponsor-approved data monitoring plan.

The majority of data collected will be inputted directly into eCRFs in the study database. Participant views on the acceptability and tolerability of the tests will be recorded on paper questionnaires and then entered at site into RCC. Participant interviews will be audio or video recorded and transcribed verbatim. Audio/video recordings and the resulting anonymised transcripts will be held in the secure HHTU instance of Box.com collaborative cloud file storage system.

The e-Consent information will be collected using DocuSign Powerforms, located within a subaccount of the HHTU instance of e-signature platform DocuSign.

RCC data will be exported and transferred to the study statistician at the University of Sheffield and the qualitative researcher at Sheffield Teaching Hospitals NHS Foundation Trust for analysis, or for Data Monitoring and Ethics Committee (DMEC) reporting in compliance with the General Data Protection Regulation Act (GDPR 2018).

### Statistical analysis

#### Blind data review

Glucocorticoid values relating to each batch of study samples analysed will undergo a quality assurance check by the study statistician before the final dataset is provided. Individual visit data will be treated as completely independent (no within-participant linkage across visits) with original participant ID numbers removed from the dataset to preserve blinding. Spaghetti plots of all timepoints within each visit will be created. Any anomalies noted will be explored and resolved prior to database lock.

#### Interim go/no go analysis

A go/no go assessment point will be scheduled approximately halfway through the recruitment phase. The independent Trial Steering Committee will consider data on retention rates and the percentage of participants completing adequate samples for cortisol exposure analysis, and will advise whether the trial should continue.

#### Final analysis

Continuous data will be summarised using n, mean, SD, median, minimum and maximum, and lower and upper quartiles will be calculated as necessary. Demographics and any baseline characteristics will be summarised overall for the safety analysis set. Medical history, which will not be coded, will be listed but not summarised.

All available data will be included in each analysis. For example, comparisons of active versus placebo treatments will include all participants providing samples following receipt of both treatments at the required timepoints, while the safety analysis will include all participant visits in which Nasacthin (active) was administered. A participant does not need to have provided data at every timepoint after every treatment to be included in the analysis, therefore it is possible that the number of participants included in each analysis may vary.

The non-inferiority in absolute serum cortisol levels at 30 min between 500 µg Nasacthin and 250 µg Synacthen will be assessed using a one-sided test, in which the difference in paired means will be compared with a prespecified non-inferiority margin of −88 nmol/L, to ensure that the response to Nasacthin is, on average, no more than 88 nmol/L lower than that to Synacthen. This non-inferiority limit (maximum clinically acceptable difference in cortisol response) is based on published within-participant repeatability of the Nasacthin test,^11^ as agreed with the MHRA, with some adjustment to allow for additional variation in the baseline cortisol due to absence of dexamethasone suppression in these participants.

The superiority outcomes (superiority of active over placebo treatment) will be assessed using change from baseline levels by comparison of the paired means using two-sided t-tests and a significance level of 5% (0.05). CIs will also be produced.

The significance level (alpha) used for the non-inferiority test has been nominally set at 0.025, but will be determined by the results of the tests of the superiority primary objectives. This analysis will include all participants providing samples 30 min after receipt of both active treatments. The closely related secondary objective to demonstrate non-inferiority in serum cortisol levels using change from baseline levels instead of absolute values will be analysed using the same methodology.

The NPA, defined as the proportion of participants for which the Nasacthin test produces a rise above a predefined threshold (430 nmol/L) in serum cortisol at 30 min, using response to Synacthen as the reference standard, will be compared with 77.5% using a one-sided exact test, and an exact CI will also be provided. The significance level (alpha) used for the test has been nominally set at 0.025, but again will be determined by the results of the tests of the superiority primary objectives.

The secondary objectives to demonstrate non-inferiority in serum cortisol and salivary cortisone levels between 500 µg Nasacthin and 250 µg Synacthen will use the same methodology as the primary non-inferiority objective; however, they will use the 60 min timepoint and, for salivary cortisone, the non-inferiority margin will be set at ±25 nmol/L. Again, this non-inferiority margin is based on the published repeatability coefficient of previous work,^11^ with adjustment for the lack of dexamethasone suppression.

For the safety analysis, all participant visits in which Nasacthin (active) was administered will be included in the analysis. Any AEs reported in association with administration of placebo or Synacthen will be summarised as a comparison. AEs, SAEs and SUSARs will be summarised by treatment received, system organ class and preferred term.

For the exploratory objective, the serum cortisol, salivary cortisol and salivary cortisone responses at 30 and 60 min after each of the active treatments will be summarised using n, mean, SD, median, minimum and maximum.

A statistical testing hierarchy will be employed to ensure the overall 5% significance level is maintained for primary and secondary outcomes. First, the superiority primary outcomes will both be tested using α=5%. Both must be significant in order to continue; if both are significant, then the non-inferiority and NPA primary outcomes will be tested using a truncated Hochberg procedure to maintain a nominal 5% significance level. If at least one of these meets the criteria for significance, then secondary outcomes for non-inferiority will be tested using Hochberg procedure to maintain a nominal 5% significance (using available alpha after testing the primary objectives).

### Pharmacodynamic (PD) data analysis

The serum cortisol and salivary glucocorticoid results will be used to estimate the total cortisol exposure, the peak cortisol response and the time of this response. The paired serum and salivary cortisol, and serum cortisol and salivary cortisone concentrations will be analysed for correlation to ensure accurate generation of the total cortisol exposure data. This PD data analysis requires a minimum of 12 participants to produce adequate samples (37.5% of the final sample size).

### Patient and public involvement and engagement

The initial study design phase included consultation with the Lay ADvice on Diabetes and Endocrine Research (LADDER) group at Sheffield Teaching Hospitals NHS Foundation Trust and patient and public involvement and engagement (PPIE) groups at Sheffield Children’s NHS Foundation Trust, specifically convening cohorts of adults and paediatric patients (and their parents) with experience of the SST and salivary tests in order to better understand the unmet needs. Their input into research design, delivery and dissemination plans, and in planning ongoing PPIE activities was also sought.

Further meetings with the LADDER group and additional Sheffield Children’s Hospital PPIE groups have been used to facilitate the codesign of trial documentation, and their input will be sought later in the study to assist with dissemination plans.

Additionally, two PPIE representatives have been identified as members of the independent Trial Steering Committee to assist with study oversight.

## Ethics and dissemination

### Regulatory approvals and trial oversight

The trial protocol and amendments have been reviewed and approved by the South Central–Hampshire A Research Ethics Committee (23/SC/0073) and by the MHRA. Sheffield Children’s NHS Foundation Trust is the study sponsor, and HHTU is responsible for trial implementation and management.

A Trial Management Group has been convened to oversee trial delivery and operations. An independent Trial Steering Committee will provide overall trial supervision on behalf of the study sponsor and project funder. A Data Monitoring and Ethics Committee (DMEC) will monitor progress, review efficacy and safety data and make recommendations on study conduct and continuation, where necessary.

### Safety considerations and AE reporting

Participants will be monitored especially closely for any hypersensitivity reaction during the first 30 min after IMP administration, and then throughout the rest of their study visit. Symptoms associated with the nasal drug administration will not be reported as AEs, unless persistent (defined as still present 30 min after IMP administration). Any AEs will be reported electronically via the study database.

The occurrence of one possibly related SAE or two possibly related severe AEs will trigger trial stopping criteria. The trial can only be restarted following discussion with the DMEC and MHRA approval of a substantial amendment.

If a possibly related SAE or severe AE occurs in an individual participant, they should not receive any subsequent IMP doses and will be withdrawn from the trial.

Emergency unblinding is available 24/7 in the event of a medical emergency.

### Dissemination

Trial results will be disseminated in peer-reviewed journals, and in conference presentations at national and international meetings. Authorship will be determined according to the International Committee of Medical Journal Editors guidelines.^18^

### Data availability

Data will not be released without sought permission from the Trial Steering Committee (TSC) before the first publication of primary endpoint analysis.

Final anonymised clinical study datasets and meta-data will be produced and stored in an appropriate format to enable discoverability and sharing. Requests for access to the dataset will be managed via UK Data Service ReSHARE’s established processes and enabled by a Data Sharing Agreement.

### Protocol version

This paper is based on Protocol version 1.4; 12 September 2024.

### Current trial status

The STARLIT-2 study opened to recruitment on 3 January 2024 and has a 12-month recruitment window. Recruitment is currently on target to finish within the specified recruitment period.

## supplementary material

10.1136/bmjopen-2024-094830 online supplemental file 1

10.1136/bmjopen-2024-094830 online supplemental file 2
