# Supplementary material for: Randomised, placebo-controlled, double-blinded, four-way crossover trial to demonstrate the comparative pharmacodynamic equivalence of a non-invasive diagnostic test for adrenal insufficiency in a healthy population: the STARLIT-2 study protocol
Source: BMJ Open. 2024 Dec 22;14(12):e094830. doi: 10.1136/bmjopen-2024-094830 (PMC11664370; doi:10.1136/bmjopen-2024-094830)
Supplement: online supplemental file 1 [file bmjopen-14-12-s001.pdf]

<< *insert site logo* >>

STARLIT-2 INFORMED CONSENT FORM

(PARENT / LEGAL GUARDIAN)

Study Title: **S**alivary **T**est of **A**drenal **R**esponse to **L**iquid **I**ntranasal **T**etracosactide – Study **2** (STARLIT-2)
Name of Researcher:
Participant ID Number:

		*Please **initial** each box below*
1.	I confirm that I have read and understood the information sheet dated XX.XX.XXXX (version X.X) for the above study.	
2.	I confirm that I have had the opportunity to consider the study information, ask questions and have had these answered satisfactorily.	
3.	I understand that my child's participation is voluntary and that I am free to withdraw my child at any time without giving any reason, without my child's medical care or legal rights being affected. I understand that any remaining samples belonging to my child will be destroyed at my request provided the samples have not been transferred to the laboratory for analysis.	
4.	I agree to my child providing five ~4 ml blood samples and nine 1 ml saliva samples during their study visit (equivalent to approximately 4 teaspoons of blood and 2 teaspoons of saliva in total per visit).	
5.	I understand that data collected during the study may be looked at by individuals from the NHS Trust research team, Hull Health Trials Unit and regulatory authorities, where it is relevant to my child taking part in this research. I give permission for these individuals to have access to my child's data.	
6.	I agree to the secure transfer, storage and use of paper and electronic personal information for the purposes of this study to Hull Health Trials Unit and the University of Sheffield.	
7.	I understand that any information that could identify my child will be kept strictly confidential and that no personal information will be included in the study report or other publication.	
8.	I understand that if there are any unexpected findings then my child will be referred to the appropriate doctor for further investigations, and I am happy for their GP to be informed of the results.	
9.	I agree to my child taking part in the above study.	

IF APPLICABLE (*for Person Of Child Bearing Potential (POCBP only)*		
10.	I agree for my child to provide a urine sample for a pregnancy test at each study visit.	
OPTIONAL (*you do not have to consent to these points in order for your child to participate in the main study*)<br>*Please **initial** the appropriate box below*		
11.	I agree to my child's General Practitioner (GP) being informed of their participation in this study.	YES<br>NO
12.	I am happy to be contacted at a later date for me and my child to take part in an interview about the study.	YES<br>NO
13.	I am happy to be contacted about my child taking part in future research.	YES<br>NO
14.	I agree that the information collected about my child may be used to support other ethically approved future research projects and may be shared anonymously with other authorised researchers conducting related research.	YES<br>NO
15.	I give consent for any of the blood and saliva samples that my child gives that are used as part of this study to be used in future ethically approved research projects.	YES<br>NO
16.	I wish to be provided with a summary of the research findings once the study is complete.	YES<br>NO

**Parent / Legal Guardian**	
Name of participant (*please print*)	
Name of participant's parent or legal guardian (*please print*)	
Relationship to participant	
Date	Signature

Person receiving consent		
Name (*please print*)	Date	Signature

When completed: 1 copy (original) Investigator Site File; 1 copy for parent / legal guardian; 1 copy for clinical record; 1 copy for HHTU
