# Supplementary material for: Randomised, placebo-controlled, double-blinded, four-way crossover trial to demonstrate the comparative pharmacodynamic equivalence of a non-invasive diagnostic test for adrenal insufficiency in a healthy population: the STARLIT-2 study protocol
Source: BMJ Open. 2024 Dec 22;14(12):e094830. doi: 10.1136/bmjopen-2024-094830 (PMC11664370; doi:10.1136/bmjopen-2024-094830)
Supplement: Supplementary file 3 [file bmjopen-2024-094830.reviewer_comments.pdf]

# PEER REVIEW HISTORY

BMJ Open publishes all reviews undertaken for accepted manuscripts. Reviewers are asked to complete a checklist review form and are provided with free text boxes to elaborate on their assessment. These free text comments are reproduced below.

## ARTICLE DETAILS

### Title (Provisional)

A randomised, placebo-controlled, double-blind, 4-way crossover trial to demonstrate the comparative pharmacodynamic equivalence of a non-invasive diagnostic test for adrenal insufficiency in a healthy population - the STARLIT-2 study protocol

### Authors

Date, Kathryn; Baster, Kathleen; Caunt, Sharon; Cohen, Judith; Debono, Miguel; Fearnside, Jane; Johnson, Trevor; Ross, Richard J; Taylor, Rosie; Elder, Charlotte Jane

---

This article was not externally reviewed at BMJ Open. Protocols that have been independently assessed prior to submission to BMJ Open are usually fast-tracked to publication on the grounds that further substantial changes will not be possible. This independent assessment will usually be external, independent review for both a substantial grant award from a non-commercial or government funder and ethics approval.
